# Controlled drainage stabilized cotton yield by enhancing photosynthesis, the antioxidant defenses and osmoregulation at reduced nitrogen fertilization

**DOI:** 10.3389/fpls.2025.1740476

**Published:** 2026-01-21

**Authors:** Yonggang Duan, Jiajia Feng, Weihan Wang, Shuaikang Liu, Dongliang Qi

**Affiliations:** 1 School of hydraulic Engineering, Zhejiang University of Water Resources and Electric Power, Hangzhou, China; 2 Zhejiang Key Laboratory of River-Lake Water Network Health Restoration, Hangzhou, China; 3 College of Life Science, Zaozhuang University, Zaozhuang, Shandong, China; 4 Engineering Research Center of Ecology and Agriculture Use of Wetland, Ministry of Education, Yangtze University, Jingzhou, Hubei, China

**Keywords:** drainage regime, *Gossypium hirsutum* L., leaf senescence characteristics, nitrogen rates, soil environment

## Abstract

Controlled drainage (CD) can improve crop yield by optimizing the soil water and nutrient environment. Nevertheless, the combined effects of reduced nitrogen fertilization and CD on crop leaf senescence characteristics is unclear. Thus, a two-year field experiment was conducted to address the effects of nitrogen fertilizer rates (280, 252, 224, and 196 kg N ha^-1^, denoted as N1, N2, N3, and N4, respectively) on the leaf area index (LAI), *SPAD* value, net photosynthetic rate (*P*_n_), activities of superoxide dismutase (SOD), peroxidases (POD), catalase (CAT), and the contents of soluble protein (SP) and malondialdehyde (MDA) in plant leaves, and the seed yield of cotton under CD and free drainage (FD). CD resulted in greater LAI, *SPAD* value, *P*_n_, SOD, POD, and CAT activities, and SP content, and smaller MDA content at the three reduced nitrogen rates, and thus obtained a relatively high seed cotton yield. The delayed leaf senescence characteristics were due to greater soil moisture and NO_3_^--^N content in the plough (0–40 cm) layer under CD. Notably, all reduced nitrogen rates significantly decreased the cottonseed yield under FD, but N2 and N3 had comparable cottonseed yields under CD. Therefore, we concluded that controlled drainage could stabilize seed cotton yield by improving photosynthesis, the antioxidant defenses and osmoregulation at 80%-90% of normal nitrogen fertilizer rate. The results also reveal the physiological mechanisms through which the drainage regime mediates crop yield under varying nitrogen rates.

## 1 Introduction

The global population is projected to reach 9.6 billion by 2050 (Jouni et al., 2018). To tackle this challenge and eliminate global hunger and poverty, food and fiber production must be doubled by the end of 2050 (FAO STAT, 2020). Nevertheless, the available agricultural land is limited. Therefore, enhancing the per-unit land area productivity is a feasible way to meet the demand of growing population. Controlled drainage (CD), a drainage water management approach, has been developed to improve crop productivity for sustainable agricultural production (Youssef et al., 2023; Zhang et al., 2025). Through the artificial elevation of the outlet, CD reduces the overall drainage volume (Ballantine and Tanner, 2013) and the loss of nitrogen and phosphorus from croplands (King et al., 2022; Youssef et al., 2023). This resulted in an enhancement of soil fertility and an improvement of soil moisture content (Dou et al., 2021; Qi and Zhu, 2023). Moreover, CD enhanced the harvesting or utilization of precipitation, leading to high crop water productivity (Tolomio and Borin, 2018; Yan et al., 2022). The increased accessibility of water and nutrients contributes to the improvement of plant physiological performance. For instance, under an optimized water and nitrogen management strategy, maize plants exhibited relatively high leaf relative water content, chlorophyll and soluble protein content, as well as a high photosynthetic rate (Qi et al., 2021). Notably, CD is characterized by high resource utilization efficiency and low environmental costs (Shedekar et al., 2021; Wesström et al., 2014; Jouni et al., 2018; Wang et al., 2020), and thus it can be considered an eco-friendly tool to save agricultural resources (Ritzema, 2016). However, previous studies have shown that the impact of CD on crop yield is inconsistent. CD has been found to increase crop yield (Wesström et al., 2014; Jouni et al., 2018; Dou et al., 2021; Qi and Zhu, 2023). Nevertheless, there were adverse effects, no effects, or uncertainties concerning the influence of CD on crop yield (Poole et al., 2013; Awale et al., 2015; Karegoudar et al., 2019; Youssef et al., 2023). Consequently, the physiological mechanisms underpinning the impacts of CD on crop yield necessitate further exploration.

Besides freshwater, nitrogen is another essential resource for crop growth and development. The nitrogen fertilization greatly affects soil water and nitrogen levels, leading to variations of shoot and root growth, and consequently the final yield (Qiu et al., 2022; Qi and Hu, 2022). Improved nitrogen fertilizer management strategies have also been developed to improve crop yield and resource use efficiency. For example, compared with conventional nitrogen fertilization, the optimized nitrogen regime (reduced and top-dressing nitrogen) enhanced soil nitrogen availability and root growth, thus obtain relatively higher yield, crop water productivity and nitrogen use efficiency in rice (Yan et al., 2022). Foliar spraying can replace soil nitrogen topdressing to realize efficient yield formation in late sown cotton production system in the Yangtze River Valley, China (Zhang et al., 2024). However, the effects of nitrogen fertilization on crop yield under CD remains largely unknown (Awale et al., 2015; Qi and Zhu, 2023).

Plants regulate their cellular metabolism and defense mechanisms in the face of drought, waterlogging, salinity, and other abiotic stresses (Ahmed et al., 2002). Superoxide dismutase (SOD), peroxidases (POD), and catalase (CAT) are three crucial protective enzymes involved in active oxygen metabolism for scavenging oxygen free radicals during plant physiological processes (Foyer and Noctor, 2000). Malondialdehyde (MDA), a stable product of membrane lipid peroxidation, reflects the degree of oxidative damage through its levels (Ren et al., 2023). Soluble proteins, the main components of various cells and organelles, its content is closely related to photosynthetic capacity (Qi et al., 2021). Chlorophyll is the most crucial and efficient pigment essential for normal photosynthesis in plants. Its content, indicated by the leaf *SPAD* value, is closely correlated with the extent of leaf senescence (Yu et al., 2023). Drought, nitrogen deficiency, and their combination reduced antioxidant enzyme activities, raised MDA contents, and accelerated chlorophyll degradation, thus speeding up leaf senescence (Li et al., 2020; Qi et al., 2021). Efficient water management (water-saving irrigation or CD) and nitrogen fertilization strategies jointly mediate plant physiological processes to boost crop yield and resource use efficiency by adjusting soil moisture and nutrient contents (Deichmann et al., 2019; Lu et al., 2021). For example, compared with traditional furrow irrigation, alternate partial root-zone irrigation enhanced soil nitrogen availability (Qi et al., 2021), which contributed to increased SOD, POD, and CAT activities, net photosynthetic rate (*P*_n_), and *SPAD* value, thereby maintaining leaf greenness (Fu et al., 2024; Li et al., 2020). Moreover, improved water management strategies (CD or alternate wetting and drying irrigation) alleviated the negative impacts of nitrogen deficiency on rice plant growth and yield (Xu et al., 2018; Wu et al., 2023). However, limited information exists on the combined effects of CD and nitrogen fertilization strategies on crop growth and development. Thus, exploring the impacts of CD and nitrogen rates on plant physiological characteristics is crucial for promoting CD regimes and nitrogen management strategies.

Cotton is the most significant fiber crop worldwide and is vital for poverty reduction. Water and nitrogen are two essential factor to determine cotton yield. For instance, compared with conventional irrigation, a mulch drip irrigation system could enhance root growth in the upper soil profile, resulting in a 30% increase in cotton yield (Zhang et al., 2017). Optimizing nitrogen application promoted the synergistic enhancement of efficient radiation utilization and leaf water utilization, thereby increasing cotton yield (Zhang et al., 2024). Moreover, the interaction between nitrogen fertilizer management and water management mediates the soil environment, thereby exerting an influence on crop growth and yield (Xu et al., 2018; Qi et al., 2020a, b; Qi and Zhu, 2023; Hu et al., 2023). Therefore, scientific management of water and nitrogen is vital for sustainable cotton production and thus has attracted wide attention (Hanrahan et al., 2021; Wang et al., 2021; Yuan et al., 2022; Liu et al., 2022). A considerable body of data exists concerning the impact of either drainage regimes or nitrogen application rates on soil mineral nitrogen content, nitrogen loss, and crop yield (Thapa et al., 2015; Karegoudar et al., 2019; Wang et al., 2020; Liu et al., 2022; Youssef et al., 2023). However, the combined effect of these factors remains ambiguous (Awale et al., 2015; Qi and Zhu, 2023); particularly the effects on leaf senescence characteristics. In addition, the Jianghan Plain in China is a crucial region for cotton production, with the planted area reaching 100,000-150,000 hectares by the end of 2020 (Liu et al., 2022). Also, in the local area, excess water is predominantly drained freely through open trenches, leading to a reduction in crop yield and waste of chemical fertilizer (King et al., 2022; Tanga et al., 2025). Therefore, exploring an improved drainage regime that delays leaf senescence under nitrogen reduction is of significant importance for the sustainable development of cotton production in this region.

The primary objectives of this study were to elucidate the impacts of CD on leaf senescence and cotton yield by examining the LAI, activities of SOD, POD, and CAT, *P*_n_, soil and *SPAD* value, soluble protein content, and MDA content under conditions of reduced nitrogen fertilizer application rates, and to expound the potential reasons. It was hypothesized that CD results in higher soil nitrogen and water contents in the plough layer by reducing nitrogen loss and water discharge via runoff, sustains normal plant growth, and thereby contributes to delay leaf senescence and consequently the stabilization of cotton yield at reduced nitrogen fertilization. The findings can offer a scientific foundation for guiding drainage and nitrogen fertilization practices in cotton cultivation within the Jianghan Plain and other regions featuring comparable environmental conditions.

## 2 Materials and methods

### 2.1 Experimental site

**Figure 1:**
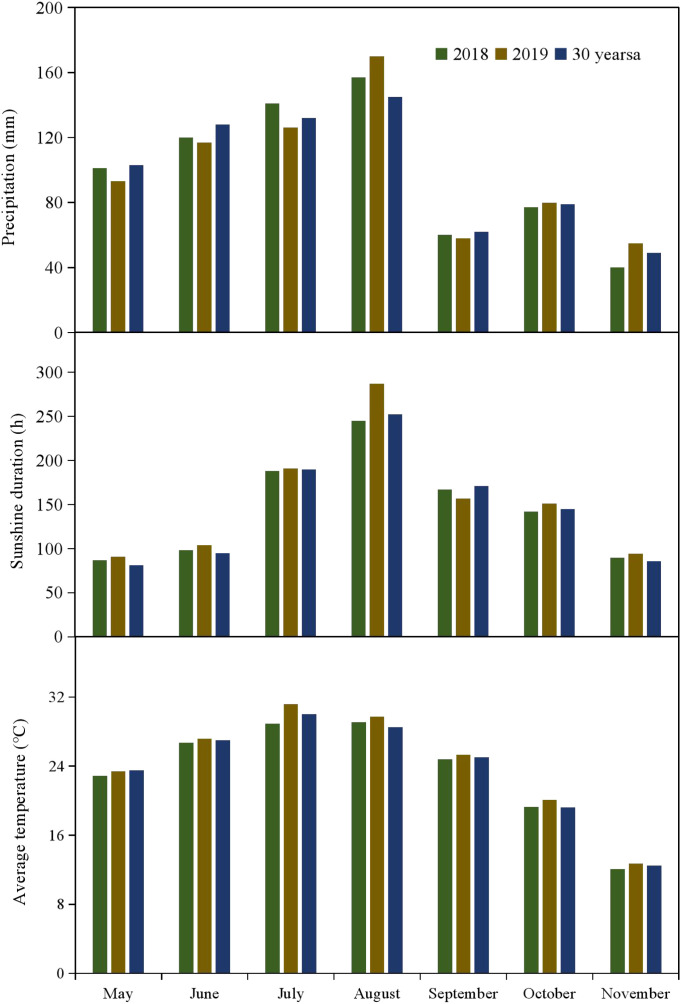
Monthly weather condition (precipitation, sunshine duration, and average temperature) during the cotton growing season in 2018 and 2019 at the experimental site.

A two-year field experiment (2018-2019) was conducted at the agricultural test station in Jingzhou City, central China (29° 26′N, 111° 15′E, 28 m above sea level). This area features a typical subtropical monsoon climate, with an average yearly rainfall of about 1,050 mm. The region enjoys a mean annual sunshine duration surpassing 1,725 hours and an average yearly temperature of 16.6°C. **Figure 1** displays monthly precipitation, mean air temperature, and sunshine duration during both cotton cultivation seasons, together with the related 30-year averages (1988-2017). According to FAO standards, the soil at the experimental site is categorized as calcareous alluvial, with a field capacity (F_c_) averaging 23.8% and a pH of 6.9. Analysis of the topsoil (0–40 cm) showed that organic matter content, total N, total phosphorus, and total potassium were 17.58, 1.25, 0.48, and 22.23 g kg^-1^, respectively. Moreover, available phosphorus, available potassium, nitrate N (NO_3_^--^N), and ammonium N (NH_4_^+^ -N) were 12.21, 85.10, 4.87, and 9.28 mg kg^-1^, respectively.

### 2.2 Experimental design

A split-plot design was utilized, where drainage regime served as the main plot and nitrogen (N) fertilizer rate acted as the sub-plot factor. Each plot covered an area of 24 m^2^ (6 m × 4 m) and was repeated three times. Two parallel drainage ditches were built in every plot, each being 6 m long, 10 cm deep, and 15 cm wide. Polyethylene film was placed within backfilled trenches to a depth of 1 m along the boundary of each plot to form a hydraulic barrier. The drainage regimes consisted of free drainage (FD) and controlled drainage (CD). In the FD regime, field ditches were handled according to natural drainage patterns, in line with locally suggested farming practices. In the CD regime, an iron sluice gate was installed at one end of the drainage ditches, and the other end was blocked with a polyvinyl chloride board to retain surface runoff within the experimental plot. The sluice gate stayed shut until the water level in the ditch reached 5 cm—a level recognized as possibly inducing waterlogging stress in cotton plants (Qi and Zhu, 2023). The sluice gate was manually operated based on visually observed water depths during rainfall occurrences. A graduated steel ruler, 20 cm long, was set up in the middle of each ditch to check the depth of collected water. Four N application rates were assessed: a reference rate of 280 kg N ha^-1^, together with three reduced levels equivalent to 90%, 80%, and 70% of the suggested nitrogen rate, namely 252, 224, and 196 kg N ha^-1^, designated as N1, N2, N3, and N4, respectively. The rate of 280 kg N ha^-1^ was taken as the recommended N level for local cotton growing according to soil test outcomes (Qi and Zhu, 2023).

### 2.3 Field management

Before sowing, calcium superphosphate (with 17% P_2_O_5_) and muriate of potash (with 60% K_2_O) were applied at rates of 529 kg ha kg^-1^ and 300 kg ha kg^-1^ respectively. Urea (46% N) was used as the nitrogen source and applied in split doses: 30% as basal fertilizer, then 30% at the bud stage and 40% at the flowering stage as topdressing. The basal fertilizers of N, P_2_O_5_, and K_2_O were applied by banding, while N topdressing was put into the planting holes. A commercial cotton variety (*Gossypium hirsutum* L.), Zhongmiansuo No.63, was used as the test material. Sowing occurred on May 10 and 12, and harvesting was carried out on November 19 and 20 in the 2018 and 2019 growing seasons respectively. Seeding furrows, each 3.5 cm deep and 5.0 cm wide, were made by an machine-drawn plough with a row spacing of 80 cm. By using a manual hill-drop sowing method, four to six seeds were placed per hill at intervals of 23.7 cm within the rows. Cotton seedlings were thinned to a density of 5.24 plants per square meter at the two-leaf stage. Each experimental plot had five rows, each 6.0 meters long and spaced 80 cm apart. Throughout the growing season, the crop depended only on natural rainfall without any additional irrigation. During both years of the study, diseases, weeds, and insect pests were well managed in all treatments.

### 2.4 Data collection

#### 2.4.1 Leaf area index and *SPAD* values

At six crucial growth stages-seeding, squaring, budding, flowering, boll setting, and maturity, measurements of leaf area and *SPAD* values were conducted. In 2018, these stages took place at 34, 55, 83, 99, 126, and 156 days after planting (DAP), respectively. For the 2019 season, the corresponding DAP values were 35, 56, 84, 100, 127, and 158 DAP. A portable area meter (LI-3050C; Li-Cor, NE, USA) was utilized to determine leaf area with green leaves gathered from eight hills. Following the method outlined by Li et al. (2020), the LAI was calculated as the overall leaf area per unit land area. *SPAD* values were measured using a handheld SPAD - 502 chlorophyll meter (Minolta Camera Co., Japan).

#### 2.4.2 Physiological measurements

Functional leaves, which are defined as the last fully-developed leaves, were sampled from three randomly-selected plants during the flowering, boll-setting, and maturity stages for measurement purposes. These measurements were performed on the same days as leaf area evaluations. Between 11:00 and 14:00 hours under clear sky conditions, *P*_n_ was measured using a portable photosynthesis system (LI-6400; Li-Cor Inc. NE, USA), with the photosynthetically active radiation kept at 1500 μmol m^-2^ s^-1^ above the canopy. Following the procedures of Ren et al. (2018), POD, SOD, and CAT were assayed using guaiacol colorimetry, nitro blue tetrazolium, and potassium nitration methods, respectively. The MDA content was quantified by the TBA method as per Du and Bramlage (1992). Soluble protein content was analyzed in accordance with the protocol devised by Mohammadkhani and Heidari (2007).

#### 2.4.3 Seed yield of cotton

In each growing season, the two central rows of cotton plants were manually harvested on four different dates: from September 20 to November 15 in 2018; and from September 20 to November 14 in 2019. After being sun dried for 15 days under natural conditions, the cottonseed was ginned when its moisture content reached ≤11%.

### 2.5 Statistical analysis

All the measured data were individually processed using a randomized complete block design (RCBD) method with the PROC GLM procedure in SAS for variance analysis. The means were compared by Duncan’s multiple range test at a significance level of *P* < 0.05. Although most of the measured N, water, and physiological parameters exhibited variation between years, there was neither year × drainage regime nor year × N interactions (**Table 1**). Therefore, we merged the data from the two different years.

**Table 1:** Analysis of variance of *SPAD* value, superoxide dismutase (SOD), peroxidases (POD), catalase (CAT), net photosynthetic rate (*P*_n_), leaf area index (LAI), *SPAD* value, malondialdehyde (MDA) and soluble protein contents under condition of drainage regimes and nitrogen management strategies interaction.

Source of variation	Degree of freedom	*SPAD*	SOD (unit. g^-1^ FW min^-1^)	POD(△OD470g^-1^ FW min^-1^)	CAT(μ mol H_2_O_2_ g^-1^FW min^-1^)	LAI (m^2^ m^-2^)	*P*_n_ (μ mol CO_2_/m ^-2^ s^-1^)	MDA (μ mol CO_2_/m ^-2^ s^-1^)	soluble protein content (mg g^−1^)
Y	1	NS	NS	NS	NS	NS	NS	NS	NS
D	1	**	**	**	**	**	**	**	**
N	3	**	**	**	**	**	**	**	**
Y×D	1	NS	NS	NS	NS	NS	NS	NS	NS
Y×N	3	NS	NS	NS	NS	NS	NS	NS	NS
D×N	3	**	**	**	**	*	**	**	*
Y×D×N	3	NS	NS	NS	NS	NS	NS	NS	NS

NS indicates statistical significance at *P* > 0.05 within a column. * and ** represents statistical significance at *P* < 0.05 and *P* < 0.01 respectively. Y, D and N represents year, drainage regime and nitrogen rate, respectively.

## 3 Results

### 3.1 Leaf area index and *SPAD* values

**Figure 2:**
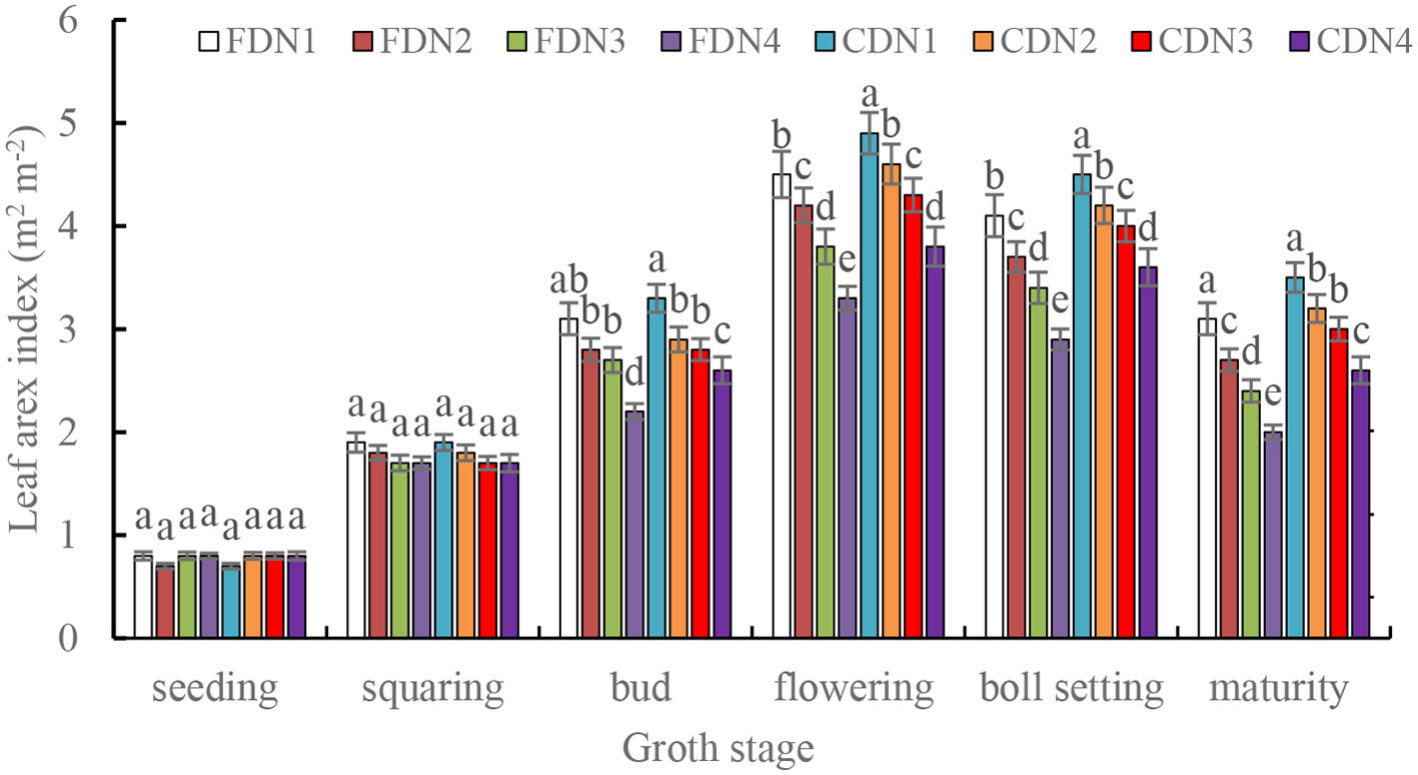
Leaf area index at the varied growth stages of cotton as affected by different nitrogen rates and drainage regimes. CD and FD represents controlled drainage and free drainage, respectively. N1, N2, N3 and N4 represent 280, 252, 224 and 196 kg N ha^-1^, respectively. Values (mean ± standard error, n = 6) are mean of 2 years and three replicates. Means within a same stage by different letters are significantly different at *p* < 0.05.

**Figure 3:**
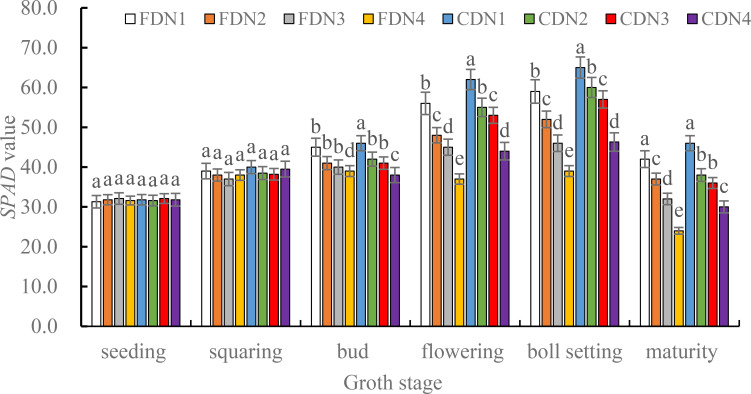
*SPAD* values of cotton leaves at the varied growth stages as affected by different nitrogen rates and drainage regimes. Note: CD and FD represents controlled drainage and free drainage, respectively. N1, N2, N3 and N4 represent 280, 252, 224 and 196 kg N ha^-1^, respectively. Values (mean ± standard error, n = 6) are mean of 2 years and three replicates. Means within a same stage by different letters are significantly different at *p* < 0.05.

The LAI at the seeding and squaring stages were comparable for the different treatments (**Figure 2**). However, the LAI at the other measured stages varied among the treatments. Compared to N1, the reduced N treatments significantly reduced LAI at the bud, flowering, boll setting and maturity stages (10.6%-35.5% smaller) under the two drainage regimes. Moreover, CD significantly increased LAI at the flowering, boll setting and maturity stages (9.5%-30.0% greater) at the reduced N rates when compared to FD. The CDN1 resulted in the greatest LAI at the flowering and boll setting stages, and the FDN4 resulted in the smallest LAI (**Figure 2**). *SPAD* values in the measured growth stages under different treatments showed similar variations compared with the LAI in the corresponding stages (**Figure 3**).

### 3.2 Activities of superoxide dismutase, peroxidases, and catalase

In all treatments, the maximum SOD, POD and CAT activities were found at the boll setting stage. Reduced N treatments significantly reduced activities of the SOD, POD and CAT at the flowering, boll setting and maturity stages (decreased by 7.8%-47.6%) under FD, while their activities at the boll setting and maturity stages were only significantly smaller in N4 (decreased by 11.1%-32.7%) under CD (**Table 2**). CD rather than FD resulted in 3.6%-31.4% higher SOD, POD and CAT activities at the three growth stages at each N rate, although the difference did not reach a significant level at N1. N4 resulted in the smallest SOD, POD and CAT activities under the two drainage regimes. The CDN1 resulted in the greatest SOD, POD and CAT activities at the three growth stages, and the FDN4 resulted in the smallest SOD, POD and CAT activities (**Table 2**).

**Table 2:** Superoxide dismutase (SOD), peroxidases (POD), and catalase (CAT) of cotton leaves at the flowering, boll setting and maturity stages as affected by different nitrogen rates and drainage regimes.

Treatment	SOD (unit. g^-1^ FW min^-1^)			POD (△OD470g^-1^ FW min^-1^)			CAT (μ mol H_2_O_2_ g^-1^FW min^-1^)		
	Flowering	Boll setting	Maturity	Flowering	Boll setting	Maturity	Flowering	Boll setting	Maturity
FDN1	409.5 ± 12.3b	445.7 ± 18.3b	410.7 ± 12.3a	181.4 ± 8.9b	215.8 ± 5.2b	145.8 ± 3.2a	14.6 ± 0.8b	18.6 ± 0.7a	12.8 ± 0.5a
FDN2	394.8 ± 8.6c	425.8 ± 11.1c	396.8 ± 10.7b	173.6 ± 7.1c	203.4 ± 8.9c	133.4 ± 1.7b	12.4 ± 1.1c	16.0 ± 0.5b	10.1 ± 0.6b
FDN3	394.4 ± 11.4c	417.8 ± 16.9c	394.6 ± 9.5b	168.1 ± 5.6c	194.1 ± 7.5d	124.1 ± 2.1c	11.5 ± 0.7c	14.5 ± 0.9b	8.5 ± 0.4c
FDN4	373.6 ± 10.5d	398.5 ± 11.1d	371.3 ± 10.2c	150.2 ± 4.2d	181.3 ± 3.9e	101.5 ± 3.4d	8.4 ± 0.4d	10.3 ± 0.5c	6.9 ± 0.5d
CDN1	422.3 ± 15.1a	454.3 ± 19.1a	412.3 ± 15.1a	189.3 ± 9.6a	226.3 ± 9.6a	150.3 ± 2.6a	17.2 ± 1.0a	19.5 ± 1.1a	13.7 ± 0.7a
CDN2	411.0 ± 10.9b	448.0 ± 28.9a	408.0 ± 11.9a	181.2 ± 7.8b	221.2 ± 10.8a	144.2 ± 4.7a	14.5 ± 0.8b	18.3 ± 1.7a	12.5 ± 1.1a
CDN3	408.3 ± 14.3b	443.7 ± 21.2a	404.4 ± 13.5a	179.6 ± 5.1b	219.8 ± 6.9a	144.3 ± 3.0a	13.1 ± 0.7b	17.9 ± 0.4a	11.4 ± 0.7a
CDN4	398.7 ± 10.6c	419.7 ± 21.5b	395.7 ± 16.1b	171.1 ± 5.7c	201.1 ± 8.6c	121.1 ± 1.9c	10.6 ± 0.5c	13.3 ± 1.9b	9.3 ± 0.7b

CD and FD represents controlled drainage and free drainage, respectively. N1, N2, N3 and N4 represent 280, 252, 224 and 196 kg N ha^-1^, respectively. Values (mean ± standard error, n = 6) are mean of 2 years and three replicates. Means within a same stage by different letters are significantly different at *p* < 0.05.

### 3.3 Net photosynthetic rate

**Figure 4:**
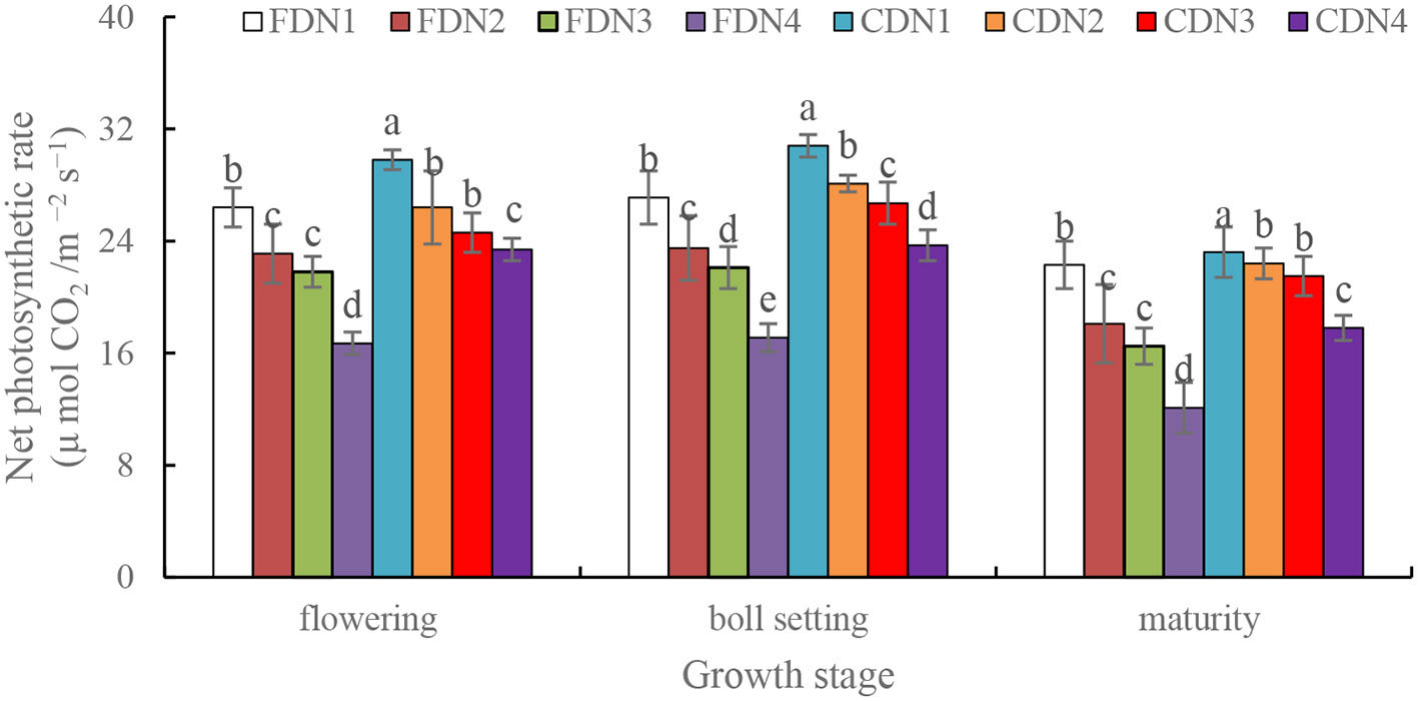
Net photosynthetic rate of cotton at the flowering, boll setting and maturity stages as affected by different nitrogen rates and drainage regimes.

Compared to normal nitrogen (N1) application, all the reduced N treatments (N2, N3, and N4) significantly reduced *P*_n_ (decreased by 10.2%-33.1%) at the flowering, boll setting and maturity stages under the two drainage regimes (**Figure 4**). Moreover, CD significantly increased *P*_n_ by 9.6%-23.7% at the flowering, boll setting and maturity stages at each N rate when compared to FD. The CDN1 resulted in the greatest *P*_n_ at the three growth stages, and the FDN4 resulted in the smallest *P*_n_ (**Figure 4**).

### 3.4 Soluble protein content

**Figure 5:**
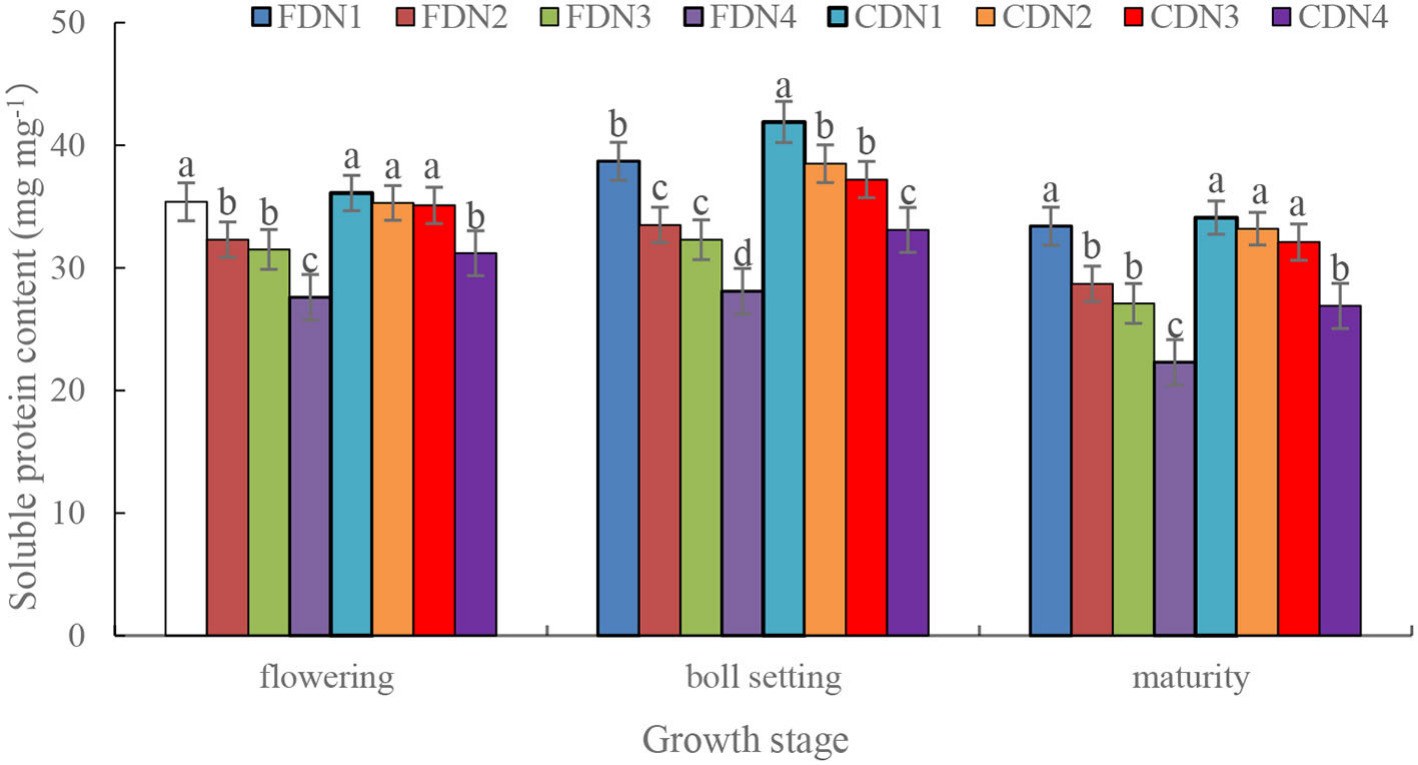
Soluble protein content of cotton leaves at the flowering, boll setting and maturity stages as affected by different nitrogen rates and drainage regimes. CD and FD represents controlled drainage and free drainage, respectively. N1, N2, N3 and N4 represent 280, 252, 224 and 196 kg N ha^-1^, respectively. Values (mean ± standard error, n = 6) are mean of 2 years and three replicates. Means within a same stage by different letters are significantly different at *p* < 0.05.

N2, N3, and N4 significantly reduced soluble protein content (decreased by 8.7%-24.5%) at the flowering, boll setting and maturity stages under FD when compared to N1 (**Figure 5**). However, N2 and N3 had a comparable soluble protein content at the flowering and maturity stage when compared to normal N rate (N1). Moreover, CD significantly increased soluble protein content by 10.1%-29.4% at the boll setting stage at each N rate when compared to FD. The CDN1 resulted in the greatest soluble protein content at the three growth stages, and the FDN4 resulted in the smallest soluble protein content (**Figure 5**).

### 3.5 Malondialdehyde content

**Figure 6:**
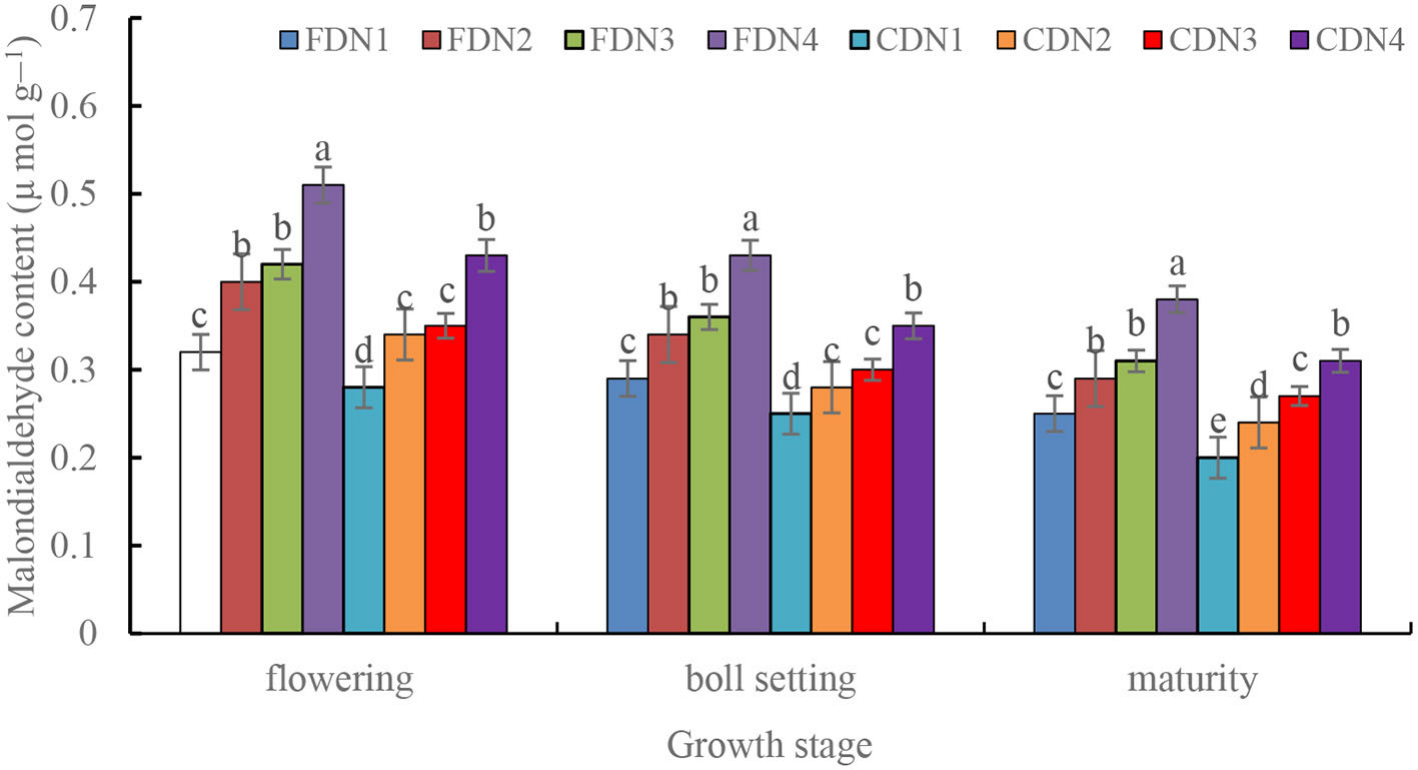
Malondialdehyde (MDA) content of cotton leaves at the flowering, boll setting and maturity stages as affected by different nitrogen rates and drainage regimes. CD and FD represents controlled drainage and free drainage, respectively. N1, N2, N3 and N4 represent 280, 252, 224 and 196 kg N ha^-1^, respectively. Values (mean ± standard error, n = 6) are mean of 2 years and three replicates. Means within a same stage by different letters are significantly different at *p* < 0.05.

All the reduced N treatments significantly increased MDA content by 9.2%-38.7% at the flowering, boll setting and maturity stages under the two drainage regimes (**Figure 6**). Moreover, CD significantly decreased MDA content by 12.1%-36.8% at the three growth stages at each N rate when compared to FD. The CDN1 resulted in the smallest MDA content at the three growth stages, and the FDN4 resulted in the greatest MDA content (**Figure 6**).

### 3.6 Seed cotton yield

**Figure 7:**
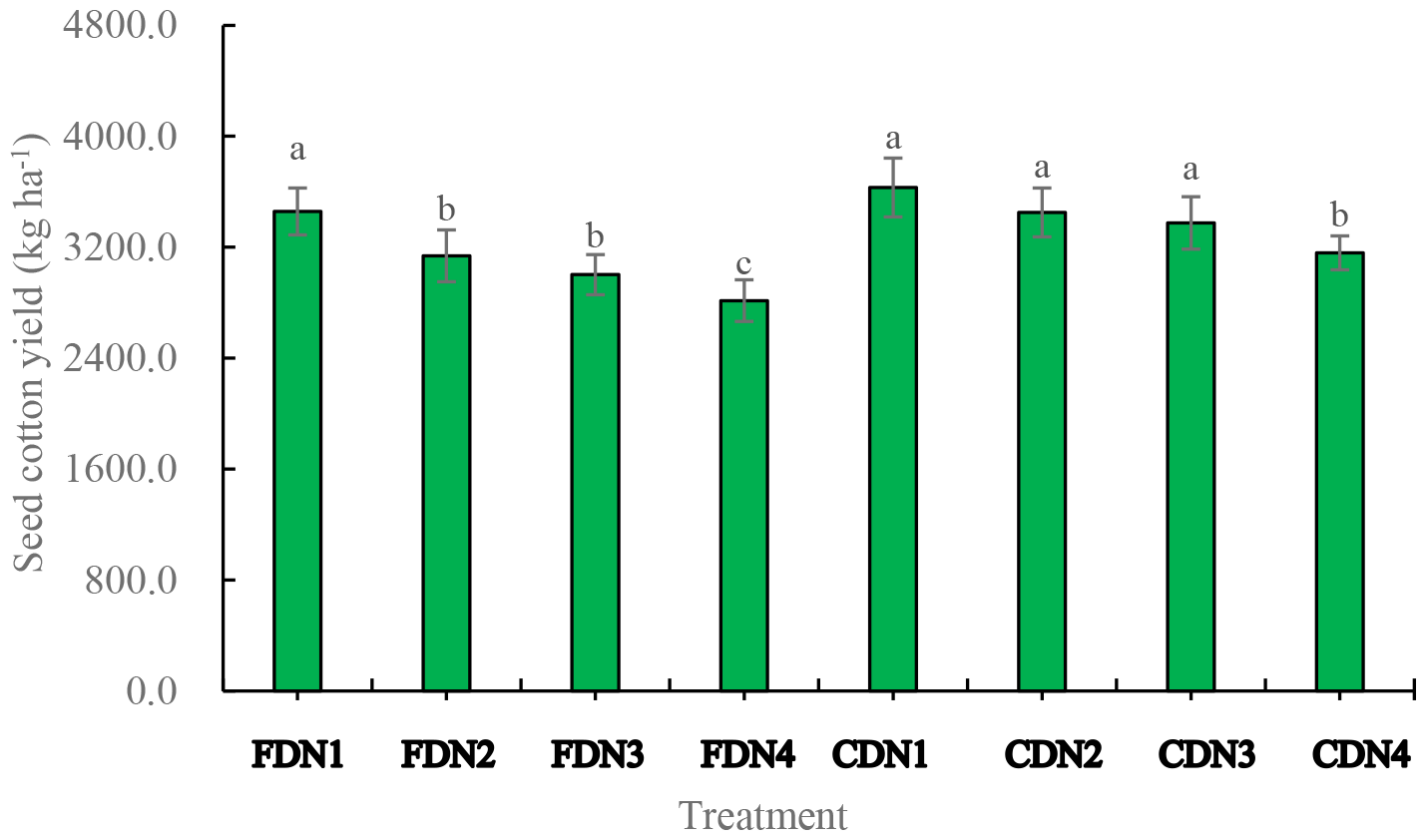
Seed cotton yield as affected by different nitrogen rates and drainage regimes. Note: CD and FD represents controlled drainage and free drainage, respectively. N1, N2, N3 and N4 represent 280, 252, 224 and 196 kg N ha^-1^, respectively. Values (mean ± standard error, n = 6) are mean of 2 years and three replicates. Means within a same stage by different letters are significantly different at *p* < 0.05.

Reductions of N application rates significantly reduced seed cotton yield by 9.2%-18.6% under FD; while only N4 significantly reduced seed cotton yield (decreased by 13.0%) under CD. FDN4 resulted in the smallest seed cotton yield (**Figure 7**).

## 4 Discussion

The influences of drainage pattern, nitrogen levels, and their interaction effects on crop yield have been assessed previously (Wu et al., 2023; Youssef et al., 2023). Moreover, our prior study has shown that a 10%-20% reduction in nitrogen fertilizer rate can keep cotton seed yield under CD (Qi and Zhu, 2023). Nevertheless, the physiological mechanism by which CD helps to stabilize cotton yield at decreased nitrogen levels is still unclear. This current research clarified that the drainage patterns and nitrogen fertilizer levels together affected the *P*_n_, LAI, *SPAD* value, activities of SOD, POD, CAT, and contents of MDA and soluble protein in cotton leaves, thus impacting cotton seed yield. Obviously, CD interacted with N2 or N3 to produce a positive interaction for delaying leaf senescence by maintaining photosynthesis, the antioxidant defense and osmoregulation, finally leading to a relatively high seed cotton yield.

### 4.1 Effects of drain regimes and reduced nitrogen rates on leaf senescence

A decrease in the LAI and *SPAD* value can mirror the leaf senescence status. The *SPAD* value offers an indirect assessment of the relative chlorophyll content, can be employed to indicate the plant’s potential ability to absorb light energy (Ren et al., 2023). The LAI can serve as an indicator of the photosynthetic potential of the canopy., thereby influencing biomass accumulation and the final crop yield (Gitelson et al., 2014). In the current study, CD generally led to greater LAI and *SPAD* values (**Figures 2**, **3**) during the bud, flowering, boll setting, and maturity stages. This implies that controlled drainage can improve functioning of cotton leaves in the middle and late growth stages. Three potential mechanisms are responsible for this phenomenon. Firstly, CD augmented the soil moisture content (**Supplementary Figure S1**) by prolonging the retention of shallow water in croplands after irrigation or precipitation (Tolomio and Borin, 2018). It also diminished the total nitrogen loss via runoff, resulting in a higher soil nitrogen availability in the plough layer during the cotton growth season (Qi and Zhu, 2023), as evidenced by the increased soil NO_3_^--^N content (**Supplementary Table S1**). The reduced total nitrogen loss through runoff was related to the significantly smaller drainage volume under CD (King et al., 2022; Tanga et al., 2025). Alternately, in low humidity (high suction) soil, the existing form of NO_3_^--^N is wholly or partially solid nitrate. In contrast, in high humidity (low suction) soil, the existing form of NO_3_^--^N is nitrate ions dissolved in the soil solution. Solid nitrate is fixed in the soil, whereas the soil solution containing nitrate ions can migrate to soil with lower moisture and high suction, driven by the matrix suction in unsaturated soil (Wang et al., 2021). During this process, the solid nitrate in the low moisture soil dissolves, leading to an increase in soil NO_3_^--^N contents in high moisture soil (Wang et al., 2021; Qi and Zhu, 2023). The improved soil moisture and nitrogen contents in the plough layer are conducive to expanding leaf size (Qi and Hu, 2022). Secondly, CD could improve the morphological characteristics of roots in oilseed sunflower (Dou et al., 2021) due to the improved soil water and nitrogen availability (Wang et al., 2021; Qi et al., 2023). The enhanced root growth resulted in various positive physiological effects mediated by abscisic acid (ABA) signaling (Liu et al., 2005). As a result, the capacity of roots to absorb soil water and nutrients was obviously enhanced (Wang et al., 2016; Zhang et al., 2021).This brought a greater leaf water content, which help to maintain large size and function of leaf (Yang et al., 2022). Thirdly, CD up-regulated the activities of SOD, POD, and CAT (**Table 2**) and down-regulated the MDA content at the post growth stages (**Figure 5**), suggesting a better reactive oxygen species scavenging ability for plants treated with CD. Moreover, CD lowering the reduction of LAI and *SPAD* values caused by decreased nitrogen rates as the enhanced soil moisture (**Supplementary Figure S1**) and NO_3_^--^N contents (**Supplementary Table S1**). Therefore, it was not surprised that the CDN1 treatment resulted in the greatest LAI and *SPAD* values (**Figures 2**, **3**). This outcome suggests that when fertility is not a limiting factor, the improved soil moisture regulation inherent in CD can be fully harnessed by the crop, leading to enhanced leaf growth. In contrast, the FDN4 treatment had the lowest NO_3_^--^N content (**Supplementary Table S1**) and smaller soil moisture content (**Supplementary Figure S1**) during the cotton growth season, corresponding to the lowest LAI and *SPAD* values in the middle and late growth stages.

The capacity to scavenge reactive oxygen species is closely associated with plant senescence (Choudhury et al., 2017). MDA interacts with proteins in the cell membrane structure and inactivates them; its content indicates the level of lipid peroxidation (Qiu et al., 2025). CD led to higher activities of SOD, POD, and CAT (**Table 2**), along with a lower MDA content (**Figure 6**), suggesting an enhanced reactive oxygen species scavenging ability under controlled drainage. This was consistent with the previously published findings of that improved water management was useful to the antioxidant defenses and osmoregulation (Hu et al., 2010). Such kind findings could also serve as the new physiological evidence to support the beneficial effects of CD on crop production, as reported in previous studies (Jouni et al., 2018; Dou et al., 2021). This was associated with a more oxygen-enriched rhizosphere, improved soil moisture, and enhanced nutrient availability under controlled drainage condition (Kaur et al., 2020). Moreover, the CDN2 and CDN3 exhibited relatively higher activities of SOD, POD, and CAT (**Table 2**), indicating better reactive oxygen species scavenging ability in plants treated with CD when N fertilizer was reduced by 10%-20%. This is parallel with the findings of Meng et al. (2023) that the sufficient water supply treatment mediated cotton growth at reduced nitrogen fertilization by enhancing photosynthesis and the activities of nitrogen metabolism enzymes. Additionally, optimal water and nitrogen management strategies up-regulate activities of antioxidant enzyme by enhancing the expression of related genes (Ozcubukcu et al., 2014). These highlight a coupling effect between water and nitrogen fertilizer, achieving both ‘regulating water with fertilizer’ and ‘promoting fertilizer with water’.

Through accumulation to augment the water-holding capacity of cells and safeguard the structure of biological membranes, the content of soluble protein is frequently employed as an indicator for detecting the abiotic stress-resistance capabilities of plants. The photosynthetic capacity can be denoted by the levels of *P*_n_ (Li et al., 2020). In the measured growth stages, CD led to an increase in *P*_n_ (**Figure 4**) and soluble protein content (**Figure 5**). This implies that controlled drainage facilitates the improvement of metabolic activities and the enhancement of photosynthetic capability, laying a solid foundation for shoot biomass accumulation (Qi et al., 2024). A high *P*_n_ was closely related with the enhanced soluble protein content, LAI, and *SPAD* values due to improved soil moisture content (Wang et al., 2016). Besides, an enhanced LAI was consistently accompanied by a higher leaf water content (Li et al., 2010). The elevated water status can suppress the production of ABA, resulting in a high stomatal conductance in leaves (Bahrun et al., 2002), and consequently, high *P*_n_ levels (Kang and Zhang, 2004). Alternatively, an obvious positive correlation existed between the activity of nitrogen-related metabolism enzymes and the root physiological characteristics in plants (Fu et al., 2024). CD optimized the rhizosphere soil environment (Youssef et al., 2023), which enhanced the root vitality (Qi et al., 2023). This phenomenon is corroborated by the relatively higher nitrogen accumulation in plants treated with CD (Qi and Zhu, 2023). Moreover, the CDN2 and CDN3 treatments had relatively high *P*_n_ and soluble protein content (**Figures 4**, **5**), suggesting that CD can stabilize the plant’s photosynthetic capability with a 10%-20% reduction in normal nitrogen fertilizer input.

### 4.2 Effects of controlled drainage regimes and reduced nitrogen rates on seed cotton yield

In this research, all the decreased N treatments led to a significant reduction in seed cotton yield under FD, whereas only the N4 significantly decreased seed cotton yield under CD (**Figure 7**). This suggests a positive interaction between CD and 10%-20% reduced nitrogen fertilization (N2 and N3) regarding cotton yield. One possible reason is that CD enhanced the soil moisture status in the plough layer (**Supplementary Figure S1**). Under conditions of ample water supply, reduced nitrogen fertilizer application was beneficial for increasing the nitrification rate and decreasing the denitrification level (Bateman and Baggs, 2005). As a result, nitrogen losses through emission, leaching, or runoff from crop fields were reduced (Ju et al., 2009; Qi and Zhu, 2023). Alternatively, at the N2 and N3 levels, CD exhibited elevated LAI, *SPAD* value, *P*_n_, SOD, POD, and CAT activities, as well as high soluble protein content (**Figures 2**-**5**; **Table 2**), while demonstrating low MDA content (**Figure 6**). These contributed to the relatively high cotton yield. Besides, CD had a comparable number of bolls, boll weight, and lint percentage at N2 and N3 (Qi and Zhu, 2023). Consistently, enhanced water management practices (such as water-saving irrigation) can partly offset the adverse effects of reduced nitrogen fertilizer rates on plant growth, thus stabilizing crop yields (Xu et al., 2018; Hu et al., 2023). This is in line with previous findings and indicates that controlled drainage adjusts the soil water environment and/or nutrient availability to improve crop yield (Jouni et al., 2018; Dou et al., 2021; Tanga et al., 2025). Nevertheless, it has been shown that drainage patterns have no impacts on the growth and yields of maize and sugar beet (Awale et al., 2015) and may even cause a reduction in maize yield (Youssef et al., 2023). These contradictions might be associated with differences in drainage patterns, weather conditions, soil fertility, crop types, etc (Kaur et al., 2020). Indeed, the reasons are still unknown and require further exploration.

In the future, the underlying mechanisms by which CD contributes to a relatively high cotton yield with a 80%-90% of normal nitrogen fertilization should be investigated from the perspective of physio-ecological characteristics (including dry weight, volume, length, surface area, oxidation activity, and the content of indole-3-acetic acid) in root and soil microbial community structure. Moreover, effects of title drainage (a more popular drainage method and it is implemented by artificially raising the outlet elevation of a subsurface drainage system) and nitrogen application rates on crop growth and development merits a further study. Furthermore, as climate patterns undergo changes, specifically with the rise in growing-season temperatures and the unpredictable distribution of precipitation, the efficacy of CD in managing soil water and nutrient storage to ensure optimal crop utilization becomes increasingly significant.

## 5 Conclusions

Controlled drainage retarded leaf senescence under a 10%-30% reduction in nitrogen fertilizer application rate by enhancing photosynthesis, the antioxidant defense system, and osmoregulation. The augmented soil moisture and NO_3_^--^N accounted for the relatively long-lasting greenness under such kind combination. Most notably, controlled drainage can be implemented without sacrificing cottonseed yield even with a 10%-20% reduction in nitrogen fertilization. This study provided the physiological mechanisms underlying controlled drainage mediates cotton yield at reduced nitrogen fertilization in humid regions.

## Data Availability

The raw data supporting the conclusions of this article will be made available by the authors, upon request.

## Supplementary material

Supplementary Figure 1 Changes in soil moisture content in the 0–80 cm soil depths during the cotton growing season under free drainage and controlled drainage. Values (mean ± standard error, n = 24) are mean of 2 years, three replicates and four nitrogen rates.

Supplementary Table 1 Nitrate nitrogen content (mg kg^-1^) in the 0–80 cm soil depths during the cotton growing season as affected by different nitrogen rates and drainage regimes. CD and FD represents controlled drainage and free drainage, respectively. N1, N2, N3 and N4 represent 280, 252, 224 and 196 kg N ha^-1^, respectively. Values (mean ± standard error, n = 6) are mean of 2 years and three replicates. Means within a same stage by different letters are significantly different at *p*  < 0.05.
