# Supplementary material for: Controlled drainage stabilized cotton yield by enhancing photosynthesis, the antioxidant defenses and osmoregulation at reduced nitrogen fertilization
Source: Front Plant Sci. 2026 Jan 21;16:1740476. doi: 10.3389/fpls.2025.1740476 (PMC12867873; doi:10.3389/fpls.2025.1740476)
Supplement: Supplementary Figure 1 — Changes in soil moisture content in the 0–80 cm soil depths during the cotton growing season under free drainage and controlled drainage. Values (mean ± standard error, n = 24) are mean of 2 years, three replicates and four nitrogen rates. [file SupplementaryFile1.docx]

**Table S1** Nitrate nitrogen content (mg kg^-1^) in the 0-80 cm soil depths during the cotton growing season as affected by different nitrogen rates and drainage regimes

| Treatment | Soil depth (cm) | | | |
| --- | --- | --- | --- | --- |
|  | 0-20 | 20-40 | 40-60 | 60-80 |
| FDN1 | 21.0±1.2b | 16.9±0.9b | 12.0±1.1a | 7.1±0.6b |
| FDN2 | 19.8±1.0c | 17.8±0.7b | 13.2±0.9a | 9.0±0.8a |
| FDN3 | 16.2±0.8d | 15.3±1.0c | 14.1±1.2a | 9.7±0.6a |
| FDN4 | 16.0±1.1d | 13.3±0.7d | 12.3±1.1a | 8.7±0.9a |
| CDN1 | 21.9±1.8a | 18.2±1.1a | 12.6±1.2a | 8.1±0.5a |
| CDN2 | 22.5±1.1a | 18.6±0.8a | 13.1±0.5a | 9.3±0.7a |
| CDN3 | 19.8±0.8c | 17.3±1.1b | 14.1±1.3a | 9.5±0.6a |
| CDN4 | 19.7±0.9c | 17.6±0.7b | 13.0±0.8a | 5.8±0.4c |

Note: CD and FD represents controlled drainage and free drainage, respectively. N1, N2, N3 and N4 represent 280, 252, 224 and 196 kg N ha^-1^, respectively. Values (mean ± standard error, n = 6) are mean of 2 years and three replicates. Means within a same stage by different letters are significantly different at *p* < 0.05.

**Figure S1**
